# Biosynthesis of Polyunsaturated Fatty Acids in *Octopus vulgaris*: Molecular Cloning and Functional Characterisation of a Stearoyl-CoA Desaturase and an Elongation of Very Long-Chain Fatty Acid 4 Protein

**DOI:** 10.3390/md15030082

**Published:** 2017-03-21

**Authors:** Óscar Monroig, Rosa de Llanos, Inmaculada Varó, Francisco Hontoria, Douglas R. Tocher, Sergi Puig, Juan C. Navarro

**Affiliations:** 1 Institute of Aquaculture, Faculty of Natural Sciences, University of Stirling, Stirling FK9 4LA, Scotland, UK; d.r.tocher@stir.ac.uk; 2 School of Applied Sciences, Edinburgh Napier University, Edinburgh EH11 4BN, Scotland, UK; R.DeLlanos@napier.ac.uk; 3 Instituto de Agroquímica y Tecnología de Alimentos (IATA-CSIC), Paterna, Valencia 46980, Spain; spuig@iata.csic.es; 4 Instituto de Acuicultura Torre de la Sal (IATS-CSIC), Ribera de Cabanes, Castellón 12595, Spain; inma@iats.csic.es (I.V.); hontoria@iats.csic.es (F.H.); jcnavarro@iats.csic.es (J.C.N.)

**Keywords:** biosynthesis, elongation of very long-chain fatty acids 4 protein, non-methylene-interrupted fatty acids, polyunsaturated fatty acids, *Octopus vulgaris*, stearoyl-CoA desaturase

## Abstract

Polyunsaturated fatty acids (PUFAs) have been acknowledged as essential nutrients for cephalopods but the specific PUFAs that satisfy the physiological requirements are unknown. To expand our previous investigations on characterisation of desaturases and elongases involved in the biosynthesis of PUFAs and hence determine the dietary PUFA requirements in cephalopods, this study aimed to investigate the roles that a stearoyl-CoA desaturase (Scd) and an elongation of very long-chain fatty acid 4 (Elovl4) protein play in the biosynthesis of essential fatty acids (FAs). Our results confirmed the *Octopus vulgaris* Scd is a ∆9 desaturase with relatively high affinity towards saturated FAs with ≥ C_18_ chain lengths. Scd was unable to desaturate 20:1*n-*15 (^∆5^20:1) suggesting that its role in the biosynthesis of non-methylene interrupted FAs (NMI FAs) is limited to the introduction of the first unsaturation at ∆9 position. Interestingly, the previously characterised ∆5 fatty acyl desaturase was indeed able to convert 20:1*n-*9 (^∆11^20:1) to ^∆5,11^20:2, an NMI FA previously detected in octopus nephridium. Additionally, Elovl4 was able to mediate the production of 24:5*n-*3 and thus can contribute to docosahexaenoic acid (DHA) biosynthesis through the Sprecher pathway. Moreover, the octopus Elovl4 was confirmed to play a key role in the biosynthesis of very long-chain (>C_24_) PUFAs.

## 1. Introduction

Cephalopods have been regarded as promising candidates for the diversification of marine aquaculture due to their great commercial interest [1]. Despite significant progress made over the last decade, culture of cephalopod species with pelagic paralarval stages like the common octopus *Octopus vulgaris* is still challenging due to the massive mortalities occurring upon the settlement phase [2]. The specific factors causing such mortalities of paralarvae remain unclear, although it has become increasingly obvious that nutritional issues associated with inadequate supply of essential nutrients such as lipids are crucial to ensure normal growth and development of *O. vulgaris* paralarvae and ultimately improve their viability [3].

**Figure 1:**
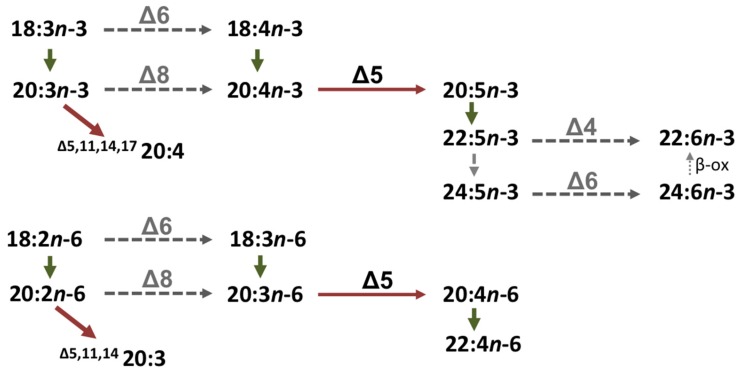
Model of biosynthetic pathways of polyunsaturated fatty acids in cephalopods. Enzymatic activities shown in the diagram are predicted from heterologous expression in yeast (*Saccharomyces cerevisiae*) of fatty acyl desaturases (red arrows) and elongation of very long-chain fatty acid (Elovl) proteins (green arrows) from *Octopus vulgaris* [6,10] and *Sepia officinalis* [20]. Dotted arrows indicate reactions that have not yet been demonstrated prior to the present study. β-ox, partial β-oxidation.

Previous investigations postulated that polyunsaturated fatty acids (PUFAs) are essential nutrients for the common octopus [4,5]. However, the specific PUFAs that satisfy the physiological requirements were not determined, partly due to the difficulties in running nutritional trials on octopus paralarvae. In order to provide insights to the endogenous capability for PUFA biosynthesis in *O. vulgaris*, we have recently conducted a series of studies aiming to identify and characterise the function of genes encoding enzymes that mediate the conversions in the PUFA biosynthetic pathways. First, we identified a fatty acyl desaturase (Fad) cDNA sequence with homology to the vertebrate Fads family [6], enzymes that participate in long-chain (C_20–24_) PUFA (LC-PUFA) biosynthetic pathways [7,8,9]. The expression of the common octopus Fad in yeast demonstrated that the enzyme was a Δ5 desaturase (Δ5 Fad) with high efficiency towards both saturated and polyunsaturated fatty acid (FA) substrates [6]. Thus, the *O. vulgaris* Δ5 Fad was able to desaturate the yeast endogenous saturated FAs 16:0 and 18:0 to the corresponding monoenes 16:1*n-*11 (^∆5^16:1) and 18:1*n-*13 (^∆5^18:1), respectively. Furthermore, the *O. vulgaris* ∆5 Fad efficiently desaturated the PUFA 20:4*n-*3 and 20:3*n-*6 to the Δ5 desaturation products eicosapentaenoic acid (EPA, 20:5*n-*3) and arachidonic acid (ARA, 20:4*n-*6), respectively (Figure 1).

A second study provided further evidence of the existence of an active PUFA biosynthetic system in the common octopus [10]. Thus, a cDNA encoding a protein with high homology to an elongation of very long-chain fatty acids (Elovl) protein was isolated [10]. Phylogenetic analysis comparing the amino acid (aa) sequence of the *O. vulgaris* Elovl with other elongases from molluscs and vertebrates clearly showed that the common octopus Elovl, as well as other putative elongases from molluscs, was grouped as a basal cluster of the vertebrate Elovl2 and Elovl5 families [10]. Consequently, such an elongase has been termed “Elovl5/2” [11] or “Elovl2/5” [12,13]. Regarding its function, the common octopus Elovl2/5 exhibited substrate specificities resembling those of vertebrate Elovl5 but not Elovl2, as it efficiently elongated C_18–20_ PUFAs [10] but had no activity towards C_22_ substrates. This was hypothesised as one of the reasons accounting for the inability of cephalopods to biosynthesise docosahexaenoic acid (DHA; 22:6*n-*3) (Figure 1) [10,11] through the so-called “Sprecher pathway” requiring the production of 24:5*n-*3 as an intermediate in the pathway [14]. Among alternative Elovl-like enzymes with a role in the biosynthesis of LC-PUFAs such as DHA in the common octopus, the Elovl4 elongase is an interesting candidate, as studies on teleost orthologues have shown that Elovl4 can efficiently catalyse the elongation of 22:5*n-*3 to 24:5*n-*3 [15,16,17,18,19], and recent studies have further demonstrated a similar elongation ability in molluscs [12].

The above studies on *O. vulgaris* [6,10], as well as those on homologous genes from the common cuttlefish *Sepia officinalis* [20], have enabled us to predict the biosynthetic pathways of PUFAs in cephalopods (Figure 1). Beyond the biosynthesis of standard PUFAs, i.e., FAs whose double bonds are always separated by a methylene group (-CH_2_-) [9], one can predict that some pathways involving the ∆5 Fad and Elovl2/5 lead to the production of so-called “non-methylene-interrupted FAs” (NMI FAs), a particular type of PUFA that had been previously reported in other molluscan classes (bivalves, gastropods), as well in sponges, echinoderms and other phyla [21,22,23]. Analyses performed in wild-caught specimens of *O. vulgaris* confirmed that the polar lipid fractions of nephridium, male gonad, eye and caecum contained NMI FAs identified as ^Δ5,11^20:2, ^Δ7,13^20:2, ^Δ5,11,14^20:3 and ^Δ7,13^22:2 [10]. From the unsaturation pattern of these compounds, it became clear that, in addition to ∆5 Fad, a further desaturase with ∆9 activity was likely involved in the NMI FA biosynthetic pathways accounting for the ∆5,9 unsaturation patterns typically found among these compounds [22,23]. The stearoyl-CoA desaturase (Scd), an enzyme that is expressed in virtually all living organisms [24], has ∆9 desaturation capability and thus appears to play a role in NMI FA biosynthesis [7].

Our overall aim is to characterise the biosynthetic pathways of PUFAs including NMI FAs in cephalopods. Using the common octopus *O. vulgaris* as model species, we herein isolated two cDNAs, namely Scd and Elovl4 sequences, and characterised their functions by heterologous expression in yeast. In order to establish the mechanisms accounting for biosynthesis of Δ5,9 dienes (NMI FA) we further investigated the roles that the herein characterised Scd and the previously reported Δ5 Fad [6] play within these pathways.

## 2. Results

### 2.1. Octopus vulgaris Scd Sequence

**Figure 2:**
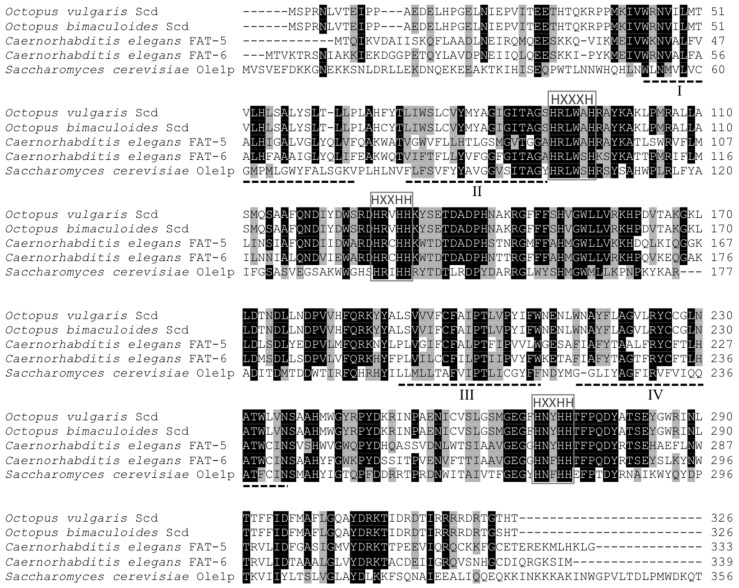
ClustalW amino acid alignment comparing the *Octopus vulgaris* stearoyl-CoA desaturase (Scd) with homologous sequences from the *Octopus bimaculoides* (XP_014788514.1), the *Caenorabditis elegans* FAT-5 (NP_507482.1) and the *C. elegans* FAT-6 (NP_001255595.1), and a portion of the *Saccharomyces cerevisiae* Ole1p sequence. Identical residues are shaded black and similar residues (using ClustalW2 default parameters) are shaded grey. The three histidine boxes (HXXXH, HXXHH and QXXHH) are highlighted with grey squares. Four (I–IV) trans-membrane domains predicted by Watts and Browse [25] are underlined with dashed lines.

The *O. vulgaris* Scd-like cDNA consisted of a 981-bp open reading frame (ORF) encoding a putative protein of 326 amino acids (aa) with a predicted molecular weight of 37.9 kDa. Its sequence was deposited in the GenBank database with the accession number JX310655. In common with other Scd proteins, the *O. vulgaris* putative Scd possessed three histidine boxes (HXXXH, HXXHH and HXXHH), four membrane-spanning regions rich in hydrophobic aa, and lacked the cytochrome b5 domain characteristic of Fads (Figure 2).

The deduced aa sequence from the common octopus Scd was 50.8%–53.4% identical to Scd sequences from vertebrates including *Homo sapiens* (NP_005054.3), *Gallus gallus* (NP_990221.1) and *Xenopus laevis* (NP_001087809.1), and 40.7% and 43.9% identical to Scd from the nematode *Caenorhabditis elegans* FAT-5 (NP_507482.1) and FAT-6 (NP_001255595.1), respectively. When compared to mollusc Scd-like protein sequences, the *O. vulgaris* Scd showed relatively high identity scores with orthologues from *Octopus bimaculoides* (99.0%) (XP_014788510.1), *Crassostrea gigas* (63.0%) (XP_011452904.1) and *Lottia gigantea* (60.7%). Importantly, identities between the newly cloned Scd and several Fads desaturases including the ∆5-like desaturase identified in *O. vulgaris* [6] were below 16.0%.

### 2.2. Octopus Elovl4-Like Sequence

**Figure 3:**
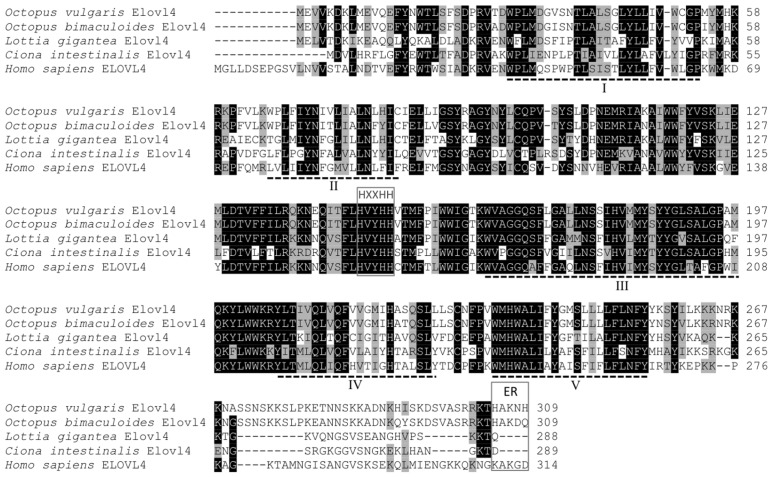
ClustalW amino acid alignment of the *Octopus vulgaris* Elovl4 with homologous sequences from *Octopus bimaculoides* (XP_014784234.1), *Lottia gigantea* (XP_009051096.1), *Ciona intestinalis* (AAV67802.1) and *Homo sapiens* (NP_073563.1). Identical residues are shaded black and similar residues (using ClustalW default parameters) are shaded grey. Indicated are the conserved histidine box motif HXXHH, five (I–V) putative membrane-spanning domains, and the putative endoplasmic reticulum (ER) retrieval signal.

The Elovl4-like cDNA consisted of an ORF of 930 bp whose deduced protein had 309 aa with a predicted molecular weight of 37.9 kDa (deposited in GenBank database with accession number KJ590963). From analogy to vertebrate orthologues [15,16,17,18], five putative transmembrane domains containing hydrophobic aa stretches can be predicted (Figure 3). The common octopus putative Elovl4 contained the histidine dideoxy-binding motif HXXHH, and the putative endoplasmic reticulum (ER) retrieval signal with a histidine (H) and lysine (K) residues at the carboxyl terminus, HXKXX (Figure 3) [26].

Comparison of the deduced aa sequence of the *O. vulgaris* Elovl4 with other orthologues revealed high identity with the *O. bimaculoides* Elovl4 (93.5%) (XP_014784234.1), with remarkable lower identity scores obtained when compared with Elovl4 sequences from *L. gigantea* (57.9%) (XP_009051096.1), *C. gigas* (41.6%) (XP_011450778.1), the sea squirt *Ciona intestinalis* (48.9%) (AAV67802.1) [27], *H. sapiens* (50.8%) (NP_073563.1), *G. gallus* (49.1%) (NP_001184238.1) and *Anolis carolinensis* (47.6%) (XP_003215742.1). Identity scores of 36.6% and 36.2% were obtained by comparing the *O. vulgaris* Elovl4 with the previously characterised Elovl2/5 from *O. vulgaris* (AFM93779.1) [10] and *S. officinalis* (AKE92956.1) [20], respectively.

### 2.3. Functional Characterisation of the Octopus Scd

**Figure 4:**
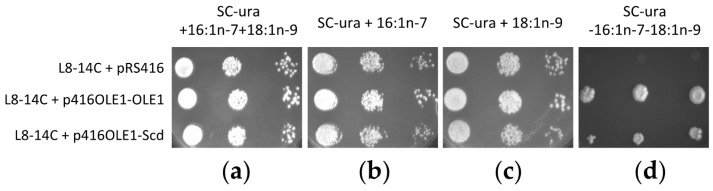
Complementation of the *Saccharomyces cerevisiae* *ole1∆* mutant strain L8-14C with the *Octopus vulgaris* stearoyl-CoA desaturase (Scd) coding region. Yeast were transformed with the pRS416 empty vector (negative control), p416OLE1-OLE1 (expressing the *S. cerevisiae* *OLE1* under the control of its own promoter) and p416OLE1-Scd (expressing the *O. vulgaris* Scd under the control of *S. cerevisiae* *OLE1* promoter) and grown in SC medium lacking uracil (SC-ura) supplemented with 16:1*n-*7 and 18:1*n-*9 (**a**), 16:1*n-*7 (**b**) or 18:1*n-*9 (**c**), or in the absence of these fatty acids (**d**).

Yeast *Saccharomyces cerevisiae* cells lacking the *OLE1* gene are unable to synthesise Δ9-monounsaturated FAs including palmitoleic acid (16:1*n-*7) and oleic acid (18:1*n-*9), which are essential for growth [28]. To address whether the *O. vulgaris* Scd was able to complement yeast *OLE1* function, an *S. cerevisiae* *ole1∆* mutant strain (L8-14C) was transformed with a plasmid expressing the octopus Scd (p416OLE1-Scd) under the control of yeast *OLE1* promoter. The *ole1∆* mutant yeast were also transformed with a plasmid expressing the *S. cerevisiae* *OLE1* (∆9 desaturase) under the control of its own promoter (p416OLE1-OLE1) as a positive control, and with empty vector (pRS416) as a negative control. All three yeast transformants were able to grow in media supplemented with at least one supplemented FA (16:1*n-*7 and/or 18:1*n-*9) (Figure 4a–c). However, only yeast cells expressing either the *S. cerevisiae* *OLE1* or the *O. vulgaris* Scd grew on medium that was not supplemented with monounsaturated FAs (Figure 4d). These results indicated that the common octopus Scd complemented the function of the yeast *OLE1* desaturase.

To analyse in more detail the complemention of yeast *ole1∆* mutants by the *O. vulgaris* Scd, we determined the FA composition of *ole1∆* yeast cells transformed with either p416OLE1-OLE1 (i.e., expressing the *S. cerevisiae* *OLE1*) or p416OLE1-Scd (i.e., expressing the *O. vulgaris* Scd), and grown in liquid medium with no exogenously supplemented FAs. Yeast expressing the *O. vulgaris* Scd grew notably slower compared to controls and formed clumps as previously described [29]. Moreover, the p416OLE1-Scd yeast contained significantly (*p* ≤ 0.05) less 16:1*n-*7 and more 16:0 than control yeast expressing the *S. cerevisiae* *OLE1*, suggesting a low affinity of the *O. vulgaris* Scd towards 16:0. On the contrary, contents of 18:1*n-*9 showed no statistical differences between yeast cells expressing Scd or *OLE1*, indicative of the octopus Scd participating in the biosynthesis of 18:1*n-*9 similar to *S. cerevisiae* Ole1p. The distinctive substrate specificities of the *O. vulgaris* Scd were further emphasised by the higher 18:1*n-*9/16:1*n-*7 ratio of yeast complemented with the *O. vulgaris* Scd compared to that of *OLE1* control yeast (Table 1).

The substrate specificities of the *O. vulgaris* Scd were further investigated through an overexpression assay using the *S. cerevisiae* strain InvSc1 (Invitrogen, Paisley, UK), possessing the endogenous Ole1p. Comparison of the FA profiles between the control yeast (i.e., transformed with the empty pYES2) and yeast expressing the *O. vulgaris* Scd (i.e., transformed with pYES2-Scd) confirmed a role of the common octopus Scd in the biosynthesis of monounsaturated FAs (Table 2). Thus, significant (*p* ≤ 0.05) increases in the contents of 18:1*n-*9 (oleic acid) in pYES2-Scd yeast compared to control yeast were observed. Parallel decreased levels of the saturated FA precursor 18:0 were detected in pYES2-Scd yeast (*p* ≤ 0.05). Additionally, other monoenes corresponding to 20:1*n-*11 and 22:1*n-*13 were detected in total lipids of InvSc1 yeast expressing the *O. vulgaris* Scd. These results confirmed that the *O. vulgaris* Scd is a Δ9 desaturase with activity towards saturated FAs with chain-lengths ≥ C_18_. In contrast, shorter FAs (≤C_16_) did not appear to be adequate substrates for the octopus Scd and, for instance, the content of 16:1*n-*7 in yeast expressing the common octopus Scd was significantly lower than that of control yeast (*p* ≤ 0.05) (Table 2).

**Figure 5:**
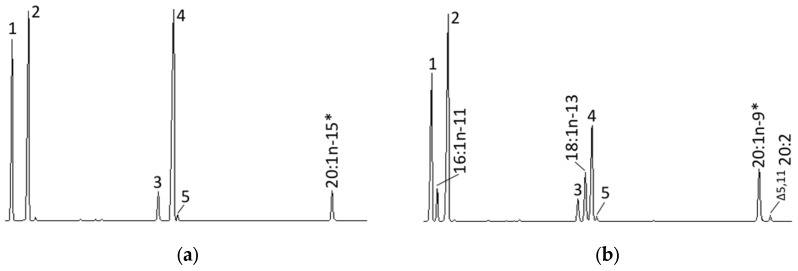
Biosynthesis of non-methylene interrupted fatty acids (FAs) in cephalopods: (**a**) Gas chromatography (GC) trace of yeast *Saccharomyces cerevisiae* expressing the *Octopus vulgaris* Scd and grown in the presence of 20:1*n-*15 (^∆5^20:1); (**b**) GC trace of yeast *S. cerevisiae* expressing the previously characterised *O. vulgaris* ∆5 Fad [6] and grown in the presence of 20:1*n-*9 (^∆11^20:1). Peaks 1-5 represent *S. cerevisiae* endogenous FAs, namely 16:0 (1), 16:1 isomers (2), 18:0 (3), 18:1*n-*9 (4) and 18:1*n-*7 (5). Peaks derived from exogenously added substrates (*) and the desaturation product ^∆5,11^20:2 (**b**) are indicated accordingly.

In order to establish the biosynthetic pathways of NMI FAs with ∆5,9 unsaturation patterns (or their derivatives such as ∆5,11) found in the common octopus lipids [10], InvSc1 yeast transformed with pYES2-Scd were grown in the presence of the monoene 20:1*n-*15 (^∆5^20:1), while InvSc1 yeast expressing the ∆5 Fad (i.e., transformed with pYES2-Fad) [6] were grown in the presence of 20:1*n-*9 (^∆11^20:1) (Figure 5). Our results showed that the *O. vulgaris* Scd was unable to desaturate the substrate ^∆5^20:1 (20:1*n-*15) to ^∆5,9^20:2 (Figure 5a), although yeast transformed with pYES2-Fad were able to produce ^∆5,11^20:2 (2.7% ± 0.2% conversion), thus confirming activity as ∆5 desaturase on ^∆11^20:1) (Figure 5b).

### 2.4. Functional Characterisation of the Octopus Elovl4

The role of the *O. vulgaris* Elovl4 in the biosynthesis of very long-chain (>C_24_) PUFAs (VLC-PUFAs) was investigated in yeast *S. cerevisiae* (strain InvSc1) expressing the Elovl4 and grown in the presence of one of either C_18_ (18:3*n-*3, 18:2*n-*6, 18:4*n-*3 and 18:3*n-*6), C_20_ (20:5*n-*3 and 20:4*n-*6), C_22_ (22:5*n-*3, 22:4*n-*6, 22:6*n-*3) or C_24_ (24:5*n-*3) PUFA substrates (Table 3). Gas Chromatography-Mass Spectrometry (GC–MS) analyses confirmed that the control yeast did not have the ability to elongate PUFAs, consistent with the previously reported lack of a PUFA elongase in *S. cerevisiae* strain InvSc1 [30]. However, the common octopus Elovl4 conferred the yeast the ability to elongate PUFAs to the corresponding elongated polyenoic products of both *n-*3 and *n-*6 series (Table 3). With the exception of DHA (22:6*n-*3), the addition of C_22_ and C_24_ PUFA substrates resulted in the production of polyenes of C_32_ products or even C_34_ when 24:5*n-*3 was used as substrate (Table 3). Indeed, the endogenous production of PUFAs with chain lengths ≥ C_26_ in yeast supplemented with exogenously supplemented C_22_ and C_24_ PUFAs allowed us to estimate the Elovl4 efficiency (as % conversion) towards potential VLC-PUFA substrates that are not commercially available. The results showed that the highest % conversions (often over 88.0%) were consistently detected on C_28_, C_30_ and C_32_ substrates (Table 3). It is noteworthy that the octopus Elovl4 was able to convert the exogenously added 20:5*n-*3 and 22:5*n-*3 to 24:5*n-*3, the substrate for DHA biosynthesis via the Sprecher pathway [14], although it showed relative low elongase activity towards DHA itself, which was marginally elongated (0.7%) to 24:6*n-*3.

## 3. Discussion

Fish and seafood are the primary sources of omega-3 long-chain (C_20–24_) PUFAs for humans [31] and this partly explains the considerable interest in elucidating the PUFA biosynthetic pathways in aquatic and marine species, particularly in farmed fish for which current trends in feed formulation are impacting the nutritional quality for human consumers [32]. Previous investigations on *O. vulgaris* [6,10,33] and *S. officinalis* [20] revealed that cephalopods possess active desaturase and elongase enzymes involved in the biosynthesis of PUFAs including NMI FAs. Using the common octopus *O. vulgaris* as model species, the present study aimed to expand our knowledge of roles that further desaturases and elongases could have on PUFA biosynthesis in cephalopods. Consequently, we characterised a stearoyl-CoA desaturase (Scd) with a putative role in the biosynthesis of NMI FAs, and an Elovl4 elongase that, in addition to its participation in the biosynthesis of VLC-PUFAs [15,16,17,18,19,34], could potentially catalyse the elongation of 22:5*n-*3 to 24:5*n-*3 required for DHA biosynthesis through the Sprecher pathway [14].

The Scd, present in virtually all living organisms [24], is an enzyme with ∆9 desaturase activity. Consistently, the newly-cloned *O. vulgaris* Scd cDNA was confirmed to encode a ∆9 desaturase that was able to operate on a range of saturated FA substrates with different chain lengths, particularly ≥ C_18_. These functions are similar to those described for two homologous sequences found in the nematode *C. elegans*, namely FAT-6 and FAT-7, which can efficiently desaturate 18:0 to 18:1*n-*9 but have lower activity towards 16:0 [25]. Interestingly, FAT-5, another Scd-like sequence existing in *C. elegans*, efficiently desaturated 16:0 to 16:1*n-*7 but had nearly undetectable activity on 18:0, and it is thus regarded as a palmitoyl-CoA-specific desaturase [25]. The functional characterisation of the *O. vulgaris* Scd and its particularly high substrate affinity towards 18:0 is consistent with 18:1*n-*9 typically appearing several folds above the levels of shorter monoenes such as 16:1*n-*7 in lipids across the Mollusca phylum [21]. Beyond its role in biosynthesis of monounsaturated FA, occurrence of NMI FAs with ∆5,9 unsaturation patterns in lipids from molluscs [22] suggested a role of Scd in the biosynthesis of this particular type of PUFA. Our results revealed that the *O. vulgaris* Scd was not able to introduce a second double bond on a ∆5 monoene such as 20:1*n-*15 (^∆5^20:1). While the possibility that cephalopod Scd can introduce ∆9 desaturations on other monoenes cannot be ruled out, these results strongly suggest that the role of Scd in the biosynthesis of NMI FAs with ∆5,9 unsaturation patterns is limited to the insertion of the first unsaturation at ∆9 position on saturated FAs. Indeed, the *O. vulgaris* ∆5 Fad, previously characterised as ∆5 desaturase [6], was able to introduce a ∆5 unsaturation on 20:1*n-*9 (^∆11^20:1) producing the NMI FA ^∆5,11^20:2. Overall, these results allow us to hypothesise that the insertion of double bonds required for NMI FA biosynthesis is initiated with a ∆9 desaturation by Scd and a subsequent ∆5 desaturation catalysed by ∆5 Fad. Compared to other molluscan classes, the occurrence of NMI FAs in cephalopods appears to be rather limited and hence the contribution of endogenous production of NMI FAs vs. dietary input is difficult to establish. Nevertheless, the mechanism of ^∆5,11^20:2 biosynthesis postulated herein aligns well with the occurrence of ^∆5,11^20:2 in the polar lipids of nephridia of adult *O. vulgaris* [10], suggesting that NMI FA biosynthesis is possible in cephalopods and likely other molluscs.

In addition to desaturases, certain Elovl enzymes play crucial roles in the biosynthesis of essential PUFAs such as EPA, ARA and DHA [9,11,26]. Whereas the previously characterised cephalopod Elovl2/5 demonstrated an ability to efficiently elongate C_18_ and C_20_ PUFA substrates [6,20], lack of elongase capability towards 22:5*n-*3 was hypothesised as a major limiting factor for DHA biosynthesis through the Sprecher pathway [6,20]. Functional characterisation of the *O. vulgaris* Elovl4 demonstrated the ability of this enzyme to contribute potentially to DHA biosynthesis by producing 24:5*n-*3, which is the substrate for ∆6 desaturation and chai*n-*shortening to produce DHA in the Sprecher pathway [14]. Such an ability of Elovl4 has been reported in fish orthologues [15,16,17,18,19]. Despite the elongation capability of *O. vulgaris* herein revealed, our overall observations on gene repertoire and function [6,10,20,33] strongly suggest that DHA is still an essential FA for cephalopods. The limited ability to biosynthesise DHA is related, rather than to an issue with elongase activity, to the apparent absence of key desaturation abilities (∆6 or ∆4) required for DHA biosynthesis according to the two aerobic pathways known in vertebrates (Figure 1). In addition to the abovementioned ∆6 desaturation required in the Sprecher pathway [14], an alternative pathway described in some teleost fish involves a ∆4 desaturation from 22:5*n-*3 to produce DHA directly (Figure 1) [9]. Molecular evidence provided by the recently published *Octopus bimaculoides* genome project [35] suggests that cephalopods possess one single Fad-like desaturase in their genome and this is likely to be a ∆5 desaturase since all the Fad-like desaturase functionally characterised not only from cephalopods such as *O. vulgaris* and *S. officinalis* [6,10] but also from bivalves [36] and gastropods [37], have ∆5 desaturase activities. In non-cephalopod molluscs, further Fad-like desaturases exist [38] but, with the exception of the ∆8 desaturase found in the scallop *Chlamys nobilis* [39], their functions have not been determined and therefore it is yet not possible to predict whether ∆6 and/or ∆4 desaturase activities exist to enable DHA biosynthesis in those species.

The *O. vulgaris* Elovl4 is also involved in the biosynthesis of VLC-PUFAs as it was able to elongate exogenously supplemented PUFAs ranging from C_18–24_ to PUFA products with chain lengths of ≥C_26_. Such conversions are largely consistent with those previously exhibited by human [34] and fish [15,16,17,18,19] Elovl4 proteins. Surprisingly, no elongation products beyond C_24_ were reported in the functional characterisation of the bivalve *C. nobilis* Elovl4 [39]. The elongation activities of the *O. vulgaris* Elovl4 estimated in the yeast expression system strongly suggested that this enzyme is particularly efficient towards C_26–30_ substrates, for which the highest % conversions were observed. Similarly, the human ELOVL4 had PUFAs of ≥C_26_ as preferred substrates for elongation, although the human ELOVL4 was able to produce up to C_38_ PUFA elongation products in mammalian cell lines [34]. Due to technical challenges in their analysis and relatively low abundance, the functions of VLC-PUFAs are not fully understood [40]. Nevertheless, the structural features of VLC-PUFAs combining those from saturated FAs at one end and those from PUFAs at the other allow unique membrane lipid conformations in photoreceptors and spermatozoa, thus suggesting important roles of VLC-PUFAs in vision and reproduction of vertebrates [40,41,42,43]. Investigations of VLC-PUFAs in cephalopods and, therefore, the herein reported activities of the *O. vulgaris* Elovl4 cannot be correlated with the presence of VLC-PUFAs in vivo. Interestingly, some very long-chain NMI FAs have been reported in nudibranchs [44,45] but, unfortunately, the potential role of Elovl4 in the biosynthesis of such compounds was not determined. Interestingly, unpublished data on tissue distribution analysis of the common octopus Elovl4 mRNA indicated that gonads and, to a lesser extent eye, were also major sites for VLC-PUFA biosynthesis in cephalopods.

In conclusion, the present study demonstrated that the common octopus *O. vulgaris* possesses an Scd with high affinity for saturated FA substrates with chain lengths of 18 carbons or longer. Its inability to introduce a double bond into ∆5 monoene strongly suggested that the role of the Scd in NMI FA biosynthesis was restricted to the introduction of the first double bond at the ∆9 position, whereas the *O. vulgaris* ∆5 Fad can introduce the second unsaturation at the ∆5 position according to the herein confirmed ability to convert 20:1*n-*9 to ^∆5,11^20:2, an NMI FA previously reported in nephridia of *O. vulgaris* adult specimens. Beyond desaturases, we could also demonstrate that *O. vulgaris* possesses an Elovl4 responsible for the biosynthesis of VLC-PUFAs.

## 4. Materials and Methods

### 4.1. Tissue Samples

Tissue samples including brain, nerve, muscle, heart, hepatopancreas, gill, caecum, eye, nephridium and gonads were obtained from the dissection of two (male and female) common octopus adult specimens as previously described [6,10]. Briefly, two (male and female) wild *O. vulgaris* adults (~1.5 kg) were maintained in seawater tanks at the facilities of the Instituto de Acuicultura Torre de la Sal; before they were cold, they were anesthetised and sacrificed by direct brain puncture. After collection, samples were immediately frozen at −80 °C until further analysis. Total RNA was extracted from octopus tissues using TriReagent^®^ (Sigma-Aldrich, Alcobendas, Spain) according to manufacturer’s instructions. Two μg of total RNA tissue samples were used for synthesis of first strand cDNA using M-MLV reverse transcriptase (Promega, Southampton, UK) primed with random hexamers.

### 4.2. Molecular Cloning of the Scd and Elovl4 cDNA Sequences

The full-length sequences of the Scd and Elovl4 cDNA were obtained as follows. The deduced aa sequences of Scd proteins from *Homo sapiens* (NP_005054.3), *Gallus gallus* (NP_990221.1), *Anolis carolinensis* (XP_003226591.1), *Caenorhabditis elegans* (FAT-7) (NP_504814.1), *Acheta domesticus* (AAK25796.1) and *Pediculus humanus* (XP_002424386.1) were aligned using BioEdit v5.0.6 (Tom Hall, Department of Microbiology, North Carolina State University, Raleigh, NC, USA). Conserved regions were used for in silico searches of mollusc expressed sequence tags (EST) using The National Center for Biotechnology Information (NCBI) tblastn tool (http://www.ncbi.nlm.nih.gov/). Processed Scd-like Expressed Sequence Tags (ESTs) from the molluscs *Lottia gigantea* (FC767047.1 and FC644199.1), *Aplysia californica* (FF074235.1, FF076278.1, EB307164.1, EB253812.1 and EB281044.1), *Limnaea stagnalis* (ES580199.1 and ES579822.1), *Crassostrea gigas* (CU997931.1 and FP006991.1), *Euprymna scolopes* (DW280836.1, DW269578.1 and DW273589.1) and *Ruditapes decussatus* (AM870139.1)*,* were aligned (Bioedit) and conserved regions used for the design of the degenerate primers UNID9F (5′-ATCACAGCTGGWGCTCAYCG-3′) and UNID9R (5′-TGGCATTGTGWGGGTCWGCATC-3′).

To clone the first fragment of the octopus Elovl4, blastn searches of mollusc ESTs were performed using the so-called “transcript 2” from *L. gigantea* that was previously identified by [6] (gi|Lotgi1|178149|) as query. Thus, additional Elovl4-like consensus sequences derived from ESTs from *A. californica* (EB285681.1, GD233360.1, EB316848.1, GD212825.1, EB325217.1, EB345626.1, EB345430.1), *Saccostrea kagaki* (AB375033.1) and *C. gigas* (HS215834.1, AM866458.1) were obtained and aligned with *L. gigantea* Elovl4-like to design the degenerate primers UNIE4F (5′-GCCAAGGCATTRTGGTGGTT-3′) and UNIE4R (5′-GTSAGRTATCKYTTCCACCA-3′).

Polymerase chain reactions (PCR) were performed with the GoTaq^®^ Green Master Mix (Promega) and using a mixture of cDNAs from brain, nerve and hepatopancreas as template. The PCR consisted of an initial denaturing step at 95 °C for 2 min, followed by 35 cycles of denaturation at 95 °C for 30 s, annealing at 50 °C for 30 s, extension at 72 °C for 40 s, followed by a final extension at 72 °C for 5 min. PCR products of approximately 180 bp (Scd) and 290 bp (Elovl4) were obtained and thereafter confirmed as positive by sequencing (DNA Sequencing Service, IBMCP-UPV, Valencia, Spain). Full-length cDNA of the octopus Scd and Elovl4 were completed by 5′ and 3′ rapid amplification of cDNA ends (RACE) PCR (FirstChoice RLM-RACE kit, Ambion, Applied Biosystems, Warrington, UK) with gene-specific primers shown in Table S1.

### 4.3. Sequence Analysis

The deduced aa sequences of the *O. vulgaris* Scd and Elovl4 ORF were compared to corresponding orthologues from other invertebrate and vertebrate species and sequence identity scores were calculated using the EMBOSS Needle Pairwise Sequence Alignment tool (http://www.ebi.ac.uk/Tools/psa/emboss_needle/). Deduced aa sequence alignments were carried out using the built-in ClustalW tool (BioEdit v7.0.9, Tom Hall, Department of Microbiology, North Carolina State University, Raleigh, NC, USA).

### 4.4. Functional Characterisation of Octopus Scd: Complementation Assay of the S. cerevisiae ole1∆ Mutant Strain L8-14C

The open reading frame (ORF) of the common octopus Scd was amplified from a mixture of cDNAs (brain, nerve and hepatopancreas) using the high fidelity *Pfu* Turbo DNA polymerase (Promega) and the primers OVD9VF and OVD9VR, containing restriction sites *Hin*dIII and *Xho*I, respectively (underlined in Table S1). PCR conditions consisted of an initial denaturing step at 95 °C for 2 min, followed by 32 cycles of denaturation at 95 °C for 30 s, annealing at 58 °C for 30 s, extension at 72 °C for 2.5 min, followed by a final extension at 72 °C for 5 min. After restriction of the PCR product, the *O. vulgaris* Scd ORF was cloned into the yeast expression vector p416TEF (a centromeric plasmid with a *URA3* selectable marker) to produce the construct p416TEF-Scd, in which octopus Scd was under the control of the yeast *TEF1* promoter. Subsequently, the promoter region of the *S. cerevisiae* *OLE1* ∆9-fatty acid desaturase gene amplified from yeast genomic DNA with the primers SCPromOLE1F and SCPromOLE1R containing *Sac*I and *Hin*dIII sites, respectively, was cloned upstream of the *O. vulgaris* Scd, replacing the *TEF1* promoter and producing the construct p416OLE1-Scd, in which octopus Scd is expressed under the control of the yeast *OLE1* promoter. As a positive control, the promoter and ORF of *S. cerevisiae* *OLE1* were amplified by PCR from yeast genomic DNA using the primers SCPromOLE1F and SCOLE1R that contained restriction sites for *Sac*I and *Xho*I, respectively (Table S1). To obtain the p416OLE1-OLE1 plasmid, the *OLE1* PCR fragment was cloned into p416TEF *Sac*I and *Xho*I restriction sites, allowing *TEF1* promoter replacement by *OLE1* promoter and ORF.

To address the common octopus Scd function we used *S. cerevisiae* L8-14C (MATa *ole1Δ::LEU2*, *leu2-3,112*, *trp1-1*, *ura3-52*, *his4*; kindly donated by Dr. Charles E. Martin), a strain whose *OLE1* gene has been deleted. Therefore, L8-14C cells lack the only yeast ∆9-fatty acid desaturase and require the supply of monounsaturated FAs including 0.5 mM palmitoleic (16:1*n-*7) and 0.5 mM oleic (18:1*n-*9) acids in the medium for growth. To address whether the octopus Scd complemented the growth defect displayed by L8-14C cells in media lacking FAs, yeast mutants transformed with pRS416 (empty vector as negative control), p416OLE1-OLE1 (positive control) or p416OLE1-Scd plasmids were grown in liquid SC medium lacking uracil (SC-ura) with supplemented FAs until exponential phase, and then assayed for growth on plates in 10-fold serial dilution drops starting at OD_600_ = 0.1. Plates were incubated for 3 days at 30 °C and photographed. To determine FA composition, four L8-14C colonies from yeast transformed with either p416OLE1-OLE1 or p416OLE1-Scd were grown in 10 mL of yeast extract peptone dextrose (YPD) broth lacking 18:1*n-*9 and 16:1*n-*7. After incubation at 30 °C for 48 h, a 5 mL aliquot of yeast culture was pelleted (1000 g for 2 min), washed twice with ddH_2_O, homogenised in chloroform/methanol (2:1, *v*/*v*) containing 0.01% butylated hydroxy toluene (BHT) [30,46], and kept at −20 °C until further analysis.

### 4.5. Functional Characterisation of Octopus Scd: Overexpression in the S. cerevisiae Strain InvSc1

Primers containing *Hin*dIII (forward) and *Xho*I (reverse) restriction sites (underlined in Table S1) OVD9VF and OVD9VR (Scd) were used to amplify the ORF of the *O. vulgaris* Scd, using the high fidelity *Pfu* Turbo DNA polymerase (Promega). Further cloning into the yeast expression vector pYES2 (Invitrogen), which contains a *GAL1* promoter that is inducible by galactose and has *URA3* as selective marker, was achieved after ligation of restricted ORF amplicons and plasmid pYES2 to produce the construct pYES2-Scd. The recombinant plasmids pYES2-Scd or pYES2 empty (negative control) were transformed into *S. cerevisiae* competent cells InvSc1 (S.c. EasyComp Transformation Kit, Invitrogen). Yeast were grown in SC-ura for 3 days.

One single yeast colony containing the pYES2-Scd or pYES2 was grown overnight at 30 °C in 5 mL of liquid SC-ura. Cell cultures were then used to inoculate 10 mL of fresh SC-ura for a final OD_600_ of 0.4. Four replicates for each construct (pYES2-Scd or pYES2) were run. Cells were grown at 30 °C for 5 h before the expression of the transgene was induced by the addition of galactose to 2% (*w*/*v*) [46]. After 48 h of galactose induction, yeast samples were collected, washed and homogenised in chloroform/methanol (2:1, *v*/*v*) containing 0.01% BHT. Samples were kept at −20 °C until further analysis.

### 4.6. Role of the Octopus Scd and Δ5 Fad in the Biosynthesis of Non-Methylene-Interrupted FAs

In order to establish the role of Scd and the previously characterised Δ5 Fad [6] in the biosynthetic pathways of ∆5,9 (or ∆5,11) non-methylene interrupted FAs (NMI FAs) we conducted the following experiment. First, yeast InvSc1 transformed with pYES2-Scd, i.e., expressing the octopus Scd, were grown in the presence of exogenously added 5-eicosenoic acid (20:1*n-*15 or ^∆5^20:1). Second, yeast InvSc1 transformed with pYES2-Fad, i.e., expressing the octopus Fads that was previously reported to exhibit ∆5-desaturase [6], were grown in the presence of 11-eicosenoic acid (20:1*n-*9 or ^∆11^20:1). Final concentration of exogenously-added substrates (^∆5^20:1 or ^∆11^20:1) was 0.75 mM. Culture conditions and yeast sample collection were performed as described above. Yeast transformed with empty pYES2 were also grown in presence of ^∆5^20:1 and ^∆11^20:1 as control treatments.

### 4.7. Functional Characterisation of Octopus Elovl4: Expression in the S. cerevisiae Strain InvSc1

The octopus Elovl4 was functionally characterised by heterologous expression in yeast *S. cerevisiae* (strain InvSc1, Invitrogen). Similarly, as described above for the Scd overexpression experiment, the octopus Elovl4 ORF was amplified with primers OVE4VF and OVE4VR containing restriction sites for *Hin*dIII and *Xho*I, respectively (Table S1) which allowed its cloning into pYES2 to produce the construct pYES2-Elovl4. Yeast InvSc1 transformed with pYES2-Elovl4 were grown in SC-ura plates. One colony was subsequently grown in SC-ura broth to produce a bulk culture that allowed us to establish subcultures of OD_600_ 0.4 in Erlenmeyer flasks that contained 5 mL of SC-ura broth and were in some cases supplemented with potential PUFA substrates for fatty acyl elongases. In order to assess the ability of the octopus Elovl4 to elongate PUFA substrates, yeast transformed with pYES2-Elovl4 were grown in the presence of one of the following PUFA substrates: 18:3*n-*3, 18:2*n-*6, 18:4*n-*3, 18:3*n-*6, 20:5*n-*3, 20:4*n-*6, 22:5*n-*3, 22:4*n-*6, 22:6*n-*3 and 24:5*n-*3. The FA substrates were added to the yeast cultures at final concentrations of 0.5 (C_18_), 0.75 (C_20_), 1.0 (C_22_) and 1.2 (C_24_) mM to compensate for decreased efficiency of uptake with increased chain length [47].

To test the ability of the octopus Elovl4 to elongate saturated FAs, yeast transformed with pYES2-Elovl4 or pYES2 (negative control) were grown in the absence of exogenously added substrates and the capability of the Elovl4 to elongate saturated FAs was estimated by comparing the saturated FA profiles of both transformants. Three different replicates for each treatment were established.

### 4.8. Fatty Acid Analysis by GC-MS

Lipid extracts [48] from the transgenic yeast were utilised for preparing fatty acid methyl esters (FAME) as previously described [6,10]. Briefly, FAME were identified and quantified using an Agilent 6850 Gas Chromatograph coupled to a 5975 series Mass Selective Detector (MSD, Agilent Technologies, Santa Clara, CA, USA). The activity of the newly cloned octopus Scd was estimated by comparing the FA profiles (expressed as % of total FAs) of control yeast with those from yeast transformed with p416OLE1-Scd (complementation experiment) or pYES2-Scd (overexpression experiment). The efficiency of the *O. vulgaris* ∆5 Fad to desaturate ^∆11^20:1 into the NMI FAs ^∆5,11^20:2 was calculated as % conversion according to the formula: (area of product/(area of product + area of substrate)) × 100. The double bond positions in FA products derived from conversion by transgenic yeast towards ^∆5^20:1 and ^∆11^20:1 was confirmed by preparing FA picolinyl ester derivatives according to the methodology described by [49] and modified according to [10]. Finally, the ability of the *O. vulgaris* Elovl4 to elongate the exogenously-added PUFA substrates (18:3*n-*3, 18:2*n-*6, 18:4*n-*3, 18:3*n-*6, 20:5*n-*3, 20:4*n-*6, 22:5*n-*3, 22:4*n-*6, 22:6*n-*3 and 24:5*n-*3) was calculated by the step-wise proportion of substrate FAs converted to elongated product as (areas of first product and longer chain products/(areas of all products with longer chain than substrate + substrate area)) × 100 [50].

### 4.9. Statistical Analyses

For the functional characterisation experiments (complementation and overexpression) of the octopus Scd, FA analyses from yeast samples were expressed as mean values ± standard deviation (*n* = 4). Similarly, the assay aiming to determine the ability of the octopus Elovl4 for elongation saturated FA was run in replicates (*n* = 3) and FA contents expressed as mean values ± standard deviation. Homogeneity of variances was checked by Barlett’s test. Comparison of FA profiles from control and yeast expressing the *O. vulgaris* Scd (complementation and overexpression experiments) or Elovl4 were compared with a Student’s *t*-test. Comparisons of the means with *p* values less or equal than 0.05 were considered significantly different. All the statistical analyses were carried out using the SPSS statistical package (SPSS Inc., Chicago, IL, USA).

### 4.10. Materials

All PUFA substrates were purchased from Nu-Chek Prep, Inc. (Elysian, MN, USA), except stearidonic acid (18:4*n-*3) from Sigma-Aldrich (Alcobendas, Spain) and tetracosapentaenoic acid (24:5*n-*3) from Larodan (Larodan Fine Chemicals AB, Malmö, Sweden). All chemicals used to prepare the *S. cerevisiae* media were from Sigma-Aldrich, except for the bacteriological agar obtained from Oxoid Ltd. (Hants, UK).

## Figures and Tables

**Table 1:** Fatty acid (FA) composition of the *S. cerevisiae* strain L8-14C transformed with the yeast-endogenous *OLE1* (SC OLE1) or stearoyl-CoA desaturase from *O. vulgaris* (OV Scd). Results are expressed as an area percentage of total fatty acids (FAs) found in transformed yeast. Different letters for each FA or ratio indicate significant differences among treatments (*t*-test, *p* ≤ 0.05).

Fatty Acid	SC OLE1	OV Scd
16:0	23.5 ± 3.6 ^b^	59.3 ± 4.9 ^a^
16:1*n-*9	0.5 ± 0.4	0.7 ± 0.7
16:1*n-*7	35.0 ± 2.2 ^a^	4.5 ± 1.0 ^b^
18:0	8.0 ± 1.7	5.6 ± 1.3
18:1*n-*9	31.8 ± 2.5	29.8 ± 6.0
18:1*n-*7	1.1 ± 0.2 ^a^	0.0 ± 0.0 ^b^
18:1*n-*9/16:1*n-*7	0.9 ± 0.1 ^b^	6.8 ± 2.0 ^a^

**Table 2:** Fatty acid (FA) composition of the *S. cerevisiae* InvSc1 transformed with either empty pYES2 vector (Control) or the common octopus Scd open reading frame (ORF). Different letters for each FA indicate significant differences among treatments (*t*-test, *p* ≤ 0.05).

Fatty Acid	Control	OV Scd
14:0	1.6 ± 0.2 ^a^	1.0 ± 0.1 ^b^
14:1*n-*5	0.5 ± 0.1	0.7 ± 0.1
16:0	25.9 ± 0.3 ^a^	22.2 ± 1.2 ^b^
16:1*n-*7	37.7 ± 0.8 ^a^	28.6 ± 3.3 ^b^
18:0	8.5 ± 0.3 ^a^	4.4 ± 0.9 ^b^
18:1*n-*9	24.1 ± 0.7 ^b^	40.9 ± 3.6 ^a^
18:1*n-*7	1.1 ± 0.1	1.1 ± 0.2
20:0	0.1 ± 0.0	0.1 ± 0.0
20:1*n-*11	N.D. ^b^	0.4 ± 0.2 ^a^
22:0	0.1 ± 0.0	0.1 ± 0.0
22:1*n-*13	N.D. ^b^	0.1 ± 0.0 ^a^

**Table 3:** Role of the *Octopus vulgaris* Elovl4 in the biosynthesis of very long-chain (>C_24_) polyunsaturated fatty acids (FAs). Conversions were calculated for each stepwise elongation according to the formula (areas of first product and longer chain products/(areas of all products with longer chain than substrate + substrate area)) × 100. The substrate FA varies as indicated in each step-wise elongation.

FA Substrate	Product	% Conversion	Elongation
18:3*n-*3	20:3*n-*3	2.8	C18→22
	22:3*n-*3	3.0	C20→22
18:2*n-*6	20:2*n-*6	1.1	C18→22
	22:2*n-*6	26.2	C20→22
18:4*n-*3	20:4*n-*3	1.0	C18→20
18:3*n-*6	20:3*n-*6	0.8	C18→20
20:5*n-*3	22:5*n-*3	1.9	C20→24
	24:5*n-*3	8.1	C22→24
20:4*n-*6	22:4*n-*6	1.0	C20→24
	24:4*n-*6	6.2	C22→24
22:5*n-*3	24:5*n-*3	4.8	C22→32
	26:5*n-*3	37.9	C24→32
	28:5*n-*3	95.0	C26→32
	30:5*n-*3	93.5	C28→32
	32:5*n-*3	51.9	C30→32
22:4*n-*6	24:4*n-*6	3.6	C22→32
	26:4*n-*6	44.5	C24→32
	28:4*n-*6	94.1	C26→32
	30:4*n-*6	91.5	C28→32
	32:4*n-*6	46.3	C30→32
22:6*n-*3	24:6*n-*3	0.7	C22→24
24:5*n-*3	26:5*n-*3	1.4	C24→34
	28:5*n-*3	88.8	C26→34
	30:5*n-*3	88.6	C28→34
	32:5*n-*3	63.2	C30→34
	34:5*n-*3	17.3	C32→34

## Supplementary Materials

The following are available online at www.mdpi.com/1660-3397/15/3/82/s1, Table S1: Primer sequences used in the present study.
