# Supplementary material for: Biosynthesis of Polyunsaturated Fatty Acids in Octopus vulgaris: Molecular Cloning and Functional Characterisation of a Stearoyl-CoA Desaturase and an Elongation of Very Long-Chain Fatty Acid 4 Protein
Source: Mar Drugs. 2017 Mar 21;15(3):82. doi: 10.3390/md15030082 (PMC5367039; doi:10.3390/md15030082)
Supplement: Supplementary file 1 [file marinedrugs-15-00082-s001.pdf]

Table S1. Sequences of the primer pairs used and accession numbers of the sequences used as references for primer design in the molecular cloning, functional characterisation and RT-PCR gene-expression analysis of the *Octopus vulgaris* Scd- and Elovl4-like cDNA sequences.

Aim	Transcript	Primer	Primer sequence	Accession No[1].
5' RACE PCR	Scd	OVD9R1	5'-TGGTGAACTCTGTGGTCTCG-3'	JX310655
		OVD9R2	5'-GGAAGCTAACAGTGCCCTCA-3'	
3' RACE PCR	Scd	OVD9F1	5'-AAAGCCAAGTTGCCAATGAG-3'	JX310655
		OVD9F2	5'-CGAGACCACAGAGTTCACCA-3'	
5' RACE PCR	Elovl4	OVE4R1	5'-ATCTGTTCGTTCTTTTGGCG-3'	KJ590963
		OVE4R2	5'-TGCTGGACCAAGTGCAGATA-3'	
3' RACE PCR	Elovl4	OVE4F1	5'-TATATGGTGGATTGGGACCA-3'	KJ590963
		OVE4F2	5'-TATCTGCACTTGGTCCAGCA-3'	
Functional characterisation in yeast	Ole1	SCPromOLE1F	5'-AGGGAGCTCAAGTCAAGGATTAGCGGATA-3'	NC_001139
		SCPromOLE1R	5'-TGGAAGCTTTGTTGTAATGTTTTAGTGCTGTT-3'	
		SCOLE1R	5'-TTCCTCGAGTTAAAAGAACTTACCAGTTTCGTAGA-3'	
	Scd	OVD9VF	5'-CCCAAGCTTAGAATGTCACCAAGAAACCT-3'	JX310655
		OVD9VR	5'-CCGCTCGAGTTAGGTGTGGGTACCAGT-3'	
Functional characterisation in yeast	Elovl4	OVE4VF	5'-CCCAAGCTTGAGGATAAAATGGAAGTAGTA-3'	KJ590963
		OVE4VR	5'-CCGCTCGAGGTTAATGATTTTTTGCATGCG-3'	

[1] GenBank (http://www.ncbi.nlm.nih.gov/)
